# Reduction in Pathogenicity in Yeast-like Fungi by Farnesol in Quail Model

**DOI:** 10.3390/ani12040489

**Published:** 2022-02-16

**Authors:** Nadezhda Sachivkina, Elena Vasilieva, Ekaterina Lenchenko, Olga Kuznetsova, Arfenia Karamyan, Alfia Ibragimova, Natalia Zhabo, Maria Molchanova

**Affiliations:** 1 Department of Microbiology and Virology, Institute of Medicine, Peoples’ Friendship University of Russia (RUDN University), 117198 Moscow, Russia; elenamatveeva07@mail.ru; 2 Department of Veterinary Medicine, Moscow State University of Food Production, 117198 Moscow, Russia; lenchenko.ekaterina@yandex.ru; 3 Department of Biochemistry, Institute of Medicine, Peoples’ Friendship University of Russia (RUDN University), 117198 Moscow, Russia; olya.k@mail.ru; 4 Department of Veterinary Medicine, Agrarian Technological Institute, Peoples’ Friendship University of Russia (RUDN University), 117198 Moscow, Russia; arfenya@mail.ru; 5 Department of General Pharmaceutical and Biomedical Technologies, Institute of Medicine, Peoples’ Friendship University of Russia (RUDN University), 117198 Moscow, Russia; an99_63@mail.ru; 6 Department of Foreign Languages, Institute of Medicine, Peoples’ Friendship University of Russia (RUDN University), 117198 Moscow, Russia; lys11@yandex.ru (N.Z.); masha013@mail.ru (M.M.)

**Keywords:** farnesol, quorum sensing (QS), *Candida albicans*, quail model, virulence

## Abstract

**Simple Summary:**

Despite the discoveries of new therapeutic antimycotics, the development of drug resistance is still the main clinical challenge in the treatment of mycoses. Data on the presence of new phytopreparations, along with the direct fungicidal effects that interfere with the resistance of fungal pathogens located in the biofilm, are of great interest. The use of these compounds as monotherapies or in combination with known antimycotics may be an effective strategy for preventing and/or destroying *Candida* biofilms found on the surface of biomedical devices and in vivo. The action mechanisms of farnesol in fungi have yet to be fully understood, but they are complex and likely include several mechanisms such as growth inhibition and apoptosis promotion.

**Abstract:**

*Candida albicans* was the first eukaryotic microorganism to exhibit quorum-sensing through the secretion of the sesquiterpene E, farnesol. This molecule is generated by dephosphorylation of farnesyl pyrophosphate in the mevalonate biosynthetic pathway in mammalian and yeast cells. Exogenous farnesol inhibits yeast-to-hyphal formation in a concentration- and time-dependent manner at the earliest stage of hyphal development. Much research has been devoted to studying the role of farnesol as an inhibitor of hyphal morphogenesis; however, little research has been published regarding the in vivo impacts of farnesol on fungal virulence and the development of *Candida* infection. While other studies have examined the impact of multiple doses of farnesol in addition to antimycotics, we hypothesize that *C. albicans* treated with a single dose of this quorum-sensing molecule could reduce fungal virulence in a quail model.

## 1. Introduction

Yeast-like fungi (YLF) of the genus *Candida* are unicellular microorganisms, aerobes, of relatively large size and rounded shape, and belong to conditionally pathogenic microorganisms. The genus *Candida* includes approximately 150 species, which are classified as deuteromycetes due to the complete absence of a sexual stage of development. From a medical point of view, seven of them are recognized as the most important disease-causing species: *C. albicans*, *C. tropicalis*, *C. krusei*, *C. kefir*, *C. glabrata*, *C. guilliermondii*, and *C. parapsilosis* [1,2]. In nature, fungi of the genus *Candida* are widespread. They live on household items and food, primarily on sugar-rich vegetables and fruits, in dairy products as well as in the internal cavities of mammals including humans [3,4,5].

Saprophytic fungi of the genus *Candida* are capable of acquiring pathogenic properties under certain conditions. In such cases, single or few elements of the fungus (blastospores), which are usually located on healthy mucous membranes, begin to multiply intensively, forming budding cells and multiple filamentous forms (pseudomycelia). The parasitic activity of the pathogen is manifested by the pronounced, rapid formation of a filamentous form. The fungus cells acquiring pathogenic properties attach to the epithelial cells of the mucous membrane, primarily to cells rich in glycogen. The fungi then invade the epithelial cells and parasitize their cytoplasm and nuclei, destroying the host cell [6,7].

In the resulting lesions of the mucous membrane, fungi produce endotoxin and a number of proteolytic, lipolytic, and saccharolytic enzymes. Factors contributing to the manifestation of pathogenic properties, invasiveness, and aggressiveness of *Candida* include, first of all, congenital and acquired immunodeficiency conditions; general infections and intoxication; endocrinopathies (diabetes mellitus plays a particularly important role among them); and the violation of the internal environment and the normal microbial “landscape” of the mucous membranes (dysbiosis) under the influence of endogenous and exogenous (e.g., corticosteroid hormones, antibiotics) influences [8,9].

In biological communities, microorganisms use various mechanisms for their communication. Depending on the cell density, bacteria, and YLF can produce QS signaling molecules (for example, secondary metabolites) that are involved in regulating gene expression and coordinating collective behaviors in their natural niche. The existence of these secondary metabolites plays a major role in the adhesion and colonization of host tissues and surfaces, morphogenesis, and biofilm development [10]. In YLF, farnesol plays a major role in the morphological transition, inhibiting the production of hyphae, depending on the concentration, while tyrosol performs the opposite function, stimulating the transition from spherical cells to the shape of the germ tube. Farnesol acts by negatively regulating hyphal-specific genes (EFG1, CPH1, and HST1) and derepressing transcriptional repressors (TUP1 and NRG1) downstream of the Ras1/cAMP/PKA and/or mitogen-activated protein (MAP) kinase-signaling cascades [11].

Farnesol is known to chemically modify cysteine residues through a process known as farnesylation, which is a specific sub-example of the more general covalent modification known as prenylation. Alteration of protein prenylation is well known as a factor in the pathology associated with bacterial quorum sensing and biofilm formation, and further mitigation of these effects has shown pharmacological promise [12,13,14]. As a result of prenylation with farnesyl or geranylgeraniol, the protein acquires hydrophobic properties and acquires the ability to interact with cell membranes or organelle membranes. Farnesyl pyrophosphate (FPP) is an intermediate in the biosynthesis of terpenes and terpenoids such as sterols and carotenoids. [15]. The effects of farnesol described in this manuscript on *C. albicans* do not differ significantly from the more general effects of prenylation observed for bacteria, which highlights common mechanisms in biology in both bacteria and fungi.

A reasonable solution is to analyze the changes that occur under the action of farnesol on an animal model. In this research, we used *C. albicans* incubated with farnesol for one hour and found that this strain can cause a less virulent infection. Our hypothesis is that farnesol could reduce *Candida albicans* pathogenicity in a quail model.

Our research focused on four investigations: (1) we analyzed the characteristic aspects of the biofilm development of this YLF strain; (2) we histopathologically analyzed the development of gastrointestinal candidiasis in quails; (3) we compared the hematological blood and serum biochemical analyses; and (4) we observed the changes in microbial composition in quail feces under the action of farnesol. These experiments confirmed related applications of farnesol and its research progression in antifungal therapy.

## 2. Materials and Methods

### 2.1. Strain

For our experiments, a standardized *C. albicans* collection strain ATCC 10231 was used. For cultivation, we used Sabouraud dextrose agar (Difco, Bordeaux, France) and agar with rice extract (M1026, HiMedia, Mumbai, India). Colonies of diurnal cultures of *C. albicans* from Sabouraud dextrose agar were washed with physiological solution (PhS) (pH 7.0). The concentration of YLF was 0.5, according to McFarland, which corresponded to 1.5 × 10^8^ cells/mL. The optical density (OD) of the biofilm was measured by the degree of binding of crystal violet (Himedia, Mumbai, India) at a wavelength of 580 nm (OD580) in an Immunochem-2100 microplate photometric analyzer (HTI, North Attleboro, MA, USA) [3].

### 2.2. Reagents

Farnesol (farnesol) (trans, trans-farnesol; Sigma-Adrich, Darmstadt, Germany), molar mass = 222.37 gr/mol, mass of the substance = 0.886 g/mL, the amount of the substance in moles = 0.886:222.37 = 0.004 M/mL, or 4000 µM/mL.

### 2.3. C. albicans Processing with Farnesol

The daily culture of YLF was washed three times with PhS, and the concentration of YLF was 0.5, according to McFarland. A total of 25 µL of farnesol 100 µM was added to 1 mL of a microbial suspension (experiment), and 25 µL of PhS was added to another test tube (control). Tubes were incubated for 1 h at 37 °C under constant shaking. After interaction with farnesol, YLF was washed three times with PhS [16,17].

### 2.4. Experimental Infection Quails with Candida albicans ATCC 10231

#### 2.4.1. Animals

**Figure 1:**
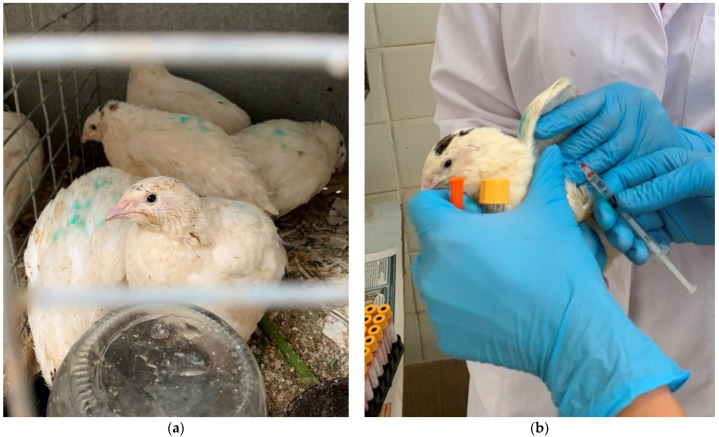
Experimental infection quails with *Candida albicans* ATCC 10231: (**a**) Birds in a cage; (**b**) Taking blood from quails for biochemical studies from the axillary vein.

Female, Texas white broiler quails (albino white pharaoh or Texas white giant) 21 days old, 30 heads, bodyweight 350–370 g, were used. Birds passed veterinary control and had all the documentation (Figure 1a). Quails were quarantined for seven days before the experiment under the supervision of a veterinarian. All animal experiments were performed in accordance with [18]. Quails were housed five birds to a cage and received food and water ad libitum, and were manipulated in accordance with the local Ethics Committee for Animal Experimentation, Peoples’ Friendship University of Russia, Moscow, Russia (protocol number 351, date 6 June 2021). The birds were divided into experimental and control groups, 15 in each. The experimental group was infected with *C. albicans* processed with farnesol, and the control group was infected with *C. albicans* without the addition of farnesol.

*C. albicans* of 1 mL was given to quails through a digestive probe once a day for five days. Five birds were euthanized every five days. The experiment lasted for 20 days: five days for infection and 15 days for the course of the experiment and observation. The control days for the studies were at five days post-infection, then at 10 and 15 days. In the tables, this is indicated as five days (experience and control), and then 10 and 15 days. Prior to euthanasia, blood samples were taken via the axillary vein in all birds and were examined histopathologically (Figure 1b) [19,20].

#### 2.4.2. Hematological Analysis of Blood Samples

Blood hematology was carried out using a CELL-DYN 3700 analyzer (Abbott, Moscow, Russia), within 45 min after blood collection, for the total red-blood-cell count (RBC), hemoglobin (HGB), and differential white-blood-cell count/mean leukocytes (LEU) including pseudoeosinophils (PSEUs), eosinophils (EOSs), monocytes (MONOs), basophils (BASs), and lymphocytes (LYMs).

#### 2.4.3. Serum Biochemical Analysis

The biochemical analysis of blood was performed on the serum within 24 h. Creatinine (CRE), aspartate aminotransferase (AST), alanine aminotransferase (ALT), alkaline phosphatase (ALP), glucose (GLU), mass concentration of cholesterol (CH) and triglycerides (TG), and total bilirubin (TBIL) estimations were carried out using Vetscan VS 2 analyzers (Abaxis, Union City, CA, USA).

### 2.5. Changes in Microbial Composition in Quail Feces

Samples were taken from the large intestine and placed in sterile test tubes for bacteriological analysis. We were interested in changes in the microbial composition in the large intestine in the experimental and control groups. After treatment with farnesol, would *Candida* become less aggressive, and were there any changes in the quantitative composition of microorganisms? For this, we prepared serial dilutions of the sample, plated the diluted suspensions, and counted the number of colony-forming units. For YLF, Sabouraud dextrose agar (BioMerieux, Paris, France) was used; for *Bifidobacterium* spp., Blaurock medium (FBIS SRCAMB, Saint-Petersburg, Russia); for *Lactobacillus* spp., MRS medium (HiMedia, Mumbai, India); for *Staphylococcus* spp., peptone–salt medium, yolk–salt agar (HiMedia, Mumbai, India); for *Streptococcus* spp., mitis-salivarius agar (HiMedia, Mumbai, India); for *Enterobacteria*, Endo’s medium, Ploskirev’s medium, and bismuth-sulfite agar (HEM, Russia); for *Clostridium* spp., clostridial agar (HiMedia, Mumbai, India); and for all others, meat-peptone agar (HEM, Moscow, Russia). The plates were incubated at 37–38 °C for 24–48 h. To create anaerobic conditions, an AnaeroJar anaerostat was used with Anaerocult gas-generating packages (Merk Ink., Darmstadt, Germany) for 72 h at a temperature of 37 °C. The pure cultures were identified using a matrix-activated laser desorption/ionization technology by MALDI Biotyper (Bruker Daltonik Inc., Billerica, MA, USA). After considering the values of the X score, which ranged from 0 to 3, values from 2 to 3 were considered successful. A result with a score of more than 2.3 was considered to be highly reliable.

The number of microorganisms in 1.0 cm^3^ of the milk sample (*C*) was calculated using the formula and expressed in logarithms with a base of 10:

C=(N/V)×K

where *N* represents the average number of colonies in 1 bacteriological cup; *V* represents the volume of suspension, which was applied when seeding the surface of the agar; and *K* represents the multiplicity of dilution.

### 2.6. Statistics

The results were analyzed using SPSS 20.0 (IBM Corp., Armonk, NY, USA). The significance of the results was determined using the Student’s t-test, and the results were considered significant when *p* < 0.05.

## 3. Results

### 3.1. Phenotypic Characteristics of C. albicans Strain Used in the Study

The *C. albicans* 10231 strain grew abundantly, forming raised, shiny-smooth, white colonies on the Sabouraud agar during 24 h at 28 °C. Under the microscope, the cells were often subspherical with a size of 3 × 4 at 4.5 μm. On day 6 at 28 °C, the culture grown on agar with the rice extract was convex and matte with an uneven edge, white in the center, with a cream color on the periphery. Microscopy found the pseudomycelium branching at an angle of 45° and yeast cells with a diameter of 3.6 μm. Terminal chlamydospores had formed.

### 3.2. Determination of Biofilm-Forming Potential of C. albicans

The optical density (OD) of *C. albicans* 10231 in the control group (0.497 ± 0.05) showed that this microorganism produced a significant biofilm (Table 1). The YLF treated with farnesol was two times less capable of forming biofilms (OD experiment 0.243 ± 0.06). Therefore, this microorganism was less effective at producing biofilm compared to the control group.

### 3.3. Experimental Infection in Quails with Candida albicans ATCC 10231

#### 3.3.1. Histopathology of Quail *Candida albicans* Infection

In the control group of birds euthanized within the first five days post-infection, swelling, weak hyperemia of the goiter mucosa, thick viscous mucus, and a delicate whitish plaque containing an abundance of budding cells and pseudomycelia were observed. In the histological sections of the goiter, oral cavity, and esophagus, fungal ingrowth into the epithelial cover of the mucous membrane had begun. On day 10 post-infection, the curd overlays were more distinct, with rounded foci. On day 15, we observed clinical signs of the disease in the form of depression, drowsiness, and poor appetite. One of the five remaining quail died prior to euthanasia. Upon autopsy, the mucous membrane of the goiter appeared “bumpier” due to the different intensity of overlays, which in sections took the form of a solid yellow-white film, and the serous membrane had folded. Delicate, loose, yellow-white overlays were noted on the mucous membrane of the oral cavity and tongue, in which multiple budding and pseudomycelial forms of the *Candida* fungus were detected. After being euthanized on day 15, the remaining four quails were found to have typical, well-marked candidiasis lesions in the anterior part of the digestive tract. In the histological sections, multiple filaments of the fungus were found growing perpendicular to the thickness of the mucous membrane.

In the experimental quails, where the YLF was treated with farnesol, we did not observe any hyperemia of the goiter mucosa in the first five days post-infection. In the histological sections of the goiter, oral cavity, and esophagus, fungal ingrowth into the epithelial cover of the mucous membrane was detected, but to a lesser extent than in the controls. On day 10 post-infection, we observed a delicate whitish plaque on the mucous membranes of the digestive tract, containing a small number of budding cells. On day 15 pre-euthanasia, clinical signs of the disease in the form of depression, drowsiness, and poor appetite were not observed in the birds, and they had all survived to this point. Upon autopsy, the mucous membrane of the goiter was found to be covered with a delicate white biofilm, in which budding *Candida albicans* were found.

Our results indicated that there was a significant difference in the form to which *C. albicans* had progressed, either as a biofilm (controls) or individual cells not connected by a matrix (experimental). In previous experiments where *Candida* was in the form of a biofilm, farnesol had been effective. However, microorganisms can also form biofilms in living models and, therefore, farnesol needs to be further tested. Our results suggest that farnesol reduced the pathogenicity of *Candida* and slowed the progression of the infection in living animals. Moreover, the YLF were less capable of forming a biofilm while in the presence of farnesol.

#### 3.3.2. Hematological and Biochemical Analysis in the Experiment and Control Groups

The results of the hematological and biochemical analyses in the experimental and control groups are summarized in Table 2 and Table 3. No statistically significant differences were observed in most of the blood hematological and biochemical parameters. However, there were differences in RBC and HGB concentration in the blood samples as these indicators were slightly higher in the control group compared to the experimental group. Small decreases in LEU, PSEU, and EOS were observed in the two groups. However, this may have to do with the advanced age of the birds, as they normally decrease slightly. The MONOs pass from the blood into the tissues and become macrophages. A decrease was observed in poisoning and severe infections. In our experiment, there was a slight decrease in monocytes between the two groups, which confirmed the severity of the candida infection. The difference in the BAS cells between the control and experimental groups was only observed at the beginning of the experiment. Only non-significant changes were observed in LYM values. In the biochemical analysis, significant increases in CRE, AST, ALT, and ALP levels were observed in the experimental group at days 10 and 15. In contrast, non-significant changes were observed in the GLU, CH, TG, and TBIL levels.

### 3.4. Changes in Microbial Composition in Quail Feces

Pronounced violations of the microbial spectrum in the large intestine were found, which were identified as dysbiosis caused by a facultative decrease in the indigenous microflora of the large intestine and an increase in conditionally pathogenic levels of *Candida*.

As shown in Table 4, the number of pathogenic *Candida* in the control group did not decrease. In the experimental group, where the *Candida* was treated with farnesol prior to infecting the quails, the amount of YLF decreased. This was due to the decreased ability of the fungi to form biofilms after treatment with farnesol. A significant difference was found in the amount of normoflora in the experimental vs. control groups. In the experimental group, *Lactobacillus* spp. and *Bifidobacterium* spp. showed better resilience due to not being hindered by pathogenic *Candida*.

**Figure 2:**
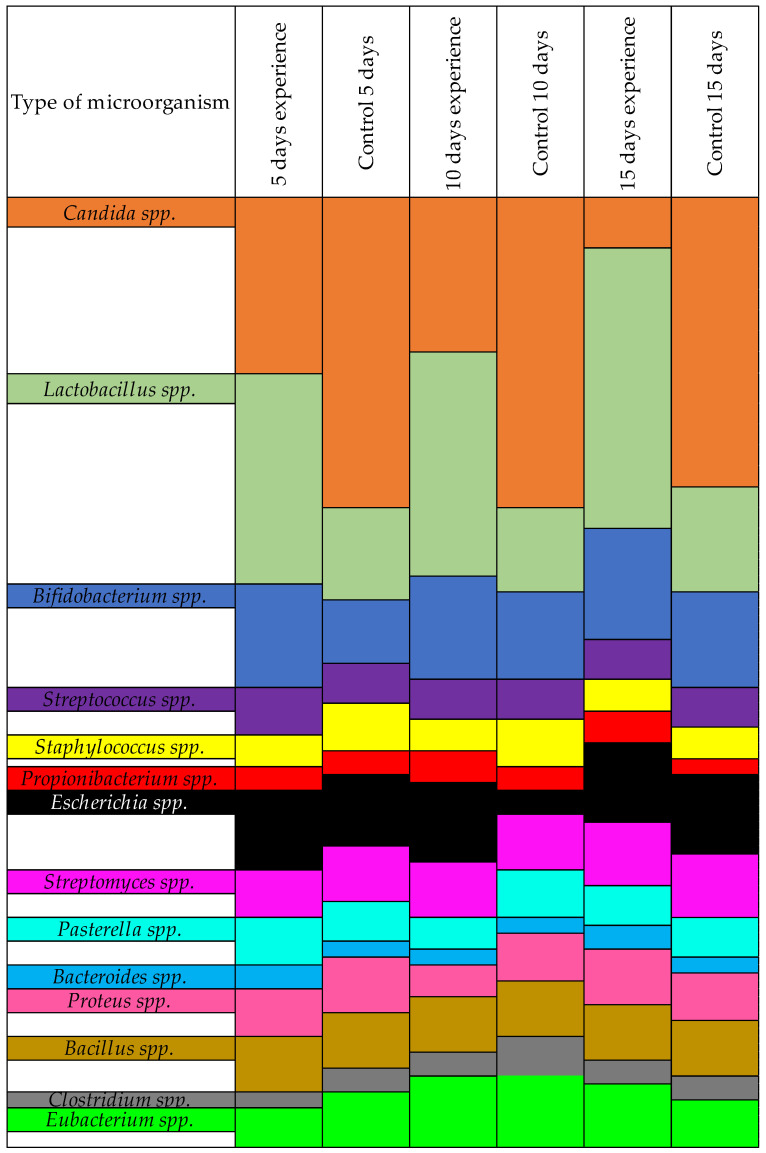
Results of the quantitative change of microorganisms in the presence of farnesol-treated *Candida*, by *%*.

The differences between the experimental and control groups were clearly visible when we recalculated our results as percentages. We assigned all microorganisms in this group at 100%, and then the shifts in microorganisms in the presence of farnesol was immediately visible, as shown in Figure 2.

## 4. Discussion

The increasing dominance of polyresistant strains of *Candida* spp. has forced scientists worldwide to consider new approaches for antimycotic drugs. A prerequisite is the safety and non-toxicity of new drugs for the patient [1,6,21]. One of the crucial factors in the virulence of *C. albicans* is its ability to switch from yeast to hyphae since the hyphal form can adhere and penetrate tissues more readily than the yeast form. The first stage in this transition is the formation of a germ tube, which is triggered by interaction with the host cells and is dependent on factors such as serum, pH, temperature, and quorum-sensing molecules (QSMs) [22,23].

The first identified QS molecule, farnesol, attracted interest, and while significant progress was made [12,24], the action mechanisms of farnesol have yet to be fully understood. Farnesol inhibited filamentation [25] including hyphal initiation [5,13,26], and filamentous growth while not disrupting their growth rates. In addition, farnesol blocked the yeast-to-hypha transition for a period of at least 6–10 h after germ-tube formation but did not block preexisting hyphal elongation [4,27]. It was reported that farnesol induced apoptosis in *C. albicans* via the accumulation of reactive oxygen species (ROS), mitochondrial degradation, and caspase activation [28].

Furthermore, farnesol and its derivatives have exhibited antibiofilm, anticancer, antitumor, and fungicidal properties [12,21,29]. The antibiofilm activity of farnesol has been described according to the time of administration during the development of a *Candida* biofilm as well as the concentration used. However, to date, the effect of farnesol and its synergy and antagonism with known antifungal drugs has not been comprehensively studied. In our experiment, we found that farnesol affected the degree of YLF resistance, which supports its potential as an antimicrobial agent [2,6,30,31].

Much research has been devoted to studying the role of farnesol as an inhibitor of hyphal morphogenesis [31,32]; however, little research has been published regarding the in vivo impacts of farnesol on fungal virulence and the development of *Candida* infection. While other studies have examined the impact of multiple doses of farnesol in addition to antimycotics [32,33], we hypothesized that *C. albicans* treated with a single dose of this quorum-sensing molecule could reduce fungal virulence in a quail model.

In our previous studies, we have shown that farnesol affected the formation of biofilms to a greater extent than planktonic cells. However, despite intensive research on farnesol over the last decade, how *C. albicans* cells sense farnesol or how this QS molecule exerts its biological effects remains unclear [34,35].

## 5. Conclusions

In summary, our results suggested that the candidiasis of the digestive tract developed to a lesser extent if treated with farnesol, and farnesol had a significant effect on the microbiological composition of the intestines. *Lactobacillus* spp. and *Bifidobacterium* spp. were present in greater concentrations when *Candida* was treated with farnesol. Given the increased patient sensitivities to existing medications reported in the literature, reducing pathogenicity and reducing biofilm formation via farnesol could be significant for the development of future therapies. This experiment may also provide a deeper understanding of the potential antifungal mechanism of QS molecules as we search for new solutions to counter infections involving *Candida*. Combined with conventional antimicrobial therapies, the therapeutic potential of this QS molecule on the virulence factors of pathogens such as *C. albicans* should be considered for biofilm-associated diseases.

## Figures and Tables

**Table 1:** Results of analyzing the optical density of biofilms.

Microorganism Culture	Optical Density (OD)	
	Control	Experiment
*C. albicans*	0.497 ± 0.05	0.243 ± 0.06

**Table 2:** Hematological parameters in the experimental and control groups of the quail models.

	Indicators							
	RBC, 10^12^	HGB, g/L	LEU, 10^9^	PSEU, %	EOS, %	MONO, %	BAS, %	LYM, %
5 days experiment	1.86 ± 0.13	85.4 ± 4.3	40.5 ± 1.9	22.2 ± 1.8	3.3 ± 0.4	3.4 ± 0.6	0.30 ± 0.05	71.5 ± 6.4
Control 5 days	1.90 ± 0.11	86.2 ± 3.9	42.1 ± 1.4	28.1 ± 2.4	3.2 ± 0.6	2.9 ± 0.8	0.42 ± 0.06	73.4 ± 4.9
10 days experiment	1.72 ± 0.12	75.4 ± 6.3	30.2 ± 0.9	24.7 ± 2.6	3.0 ± 0.4	2.4 ± 0.7	0.36 ± 0.08	79.0 ± 6.5
Control 10 days	1.84 ± 0.11	82.7 ± 5.8	38.5 ± 1.7	20.0 ± 1.9	3.5 ± 0.7	2.5 ± 0.7	0.38 ± 0.11	68.4 ± 7.1
15 days experiment	1.69 ± 0.15	72.9 ± 5.1	34.5 ± 2.1	26.5 ± 2.8	2.8 ± 0.8	2.0 ± 0.6	0.36 ± 0.05	77.6 ± 5.4
Control 15 days	1.87 ± 0.13	83.1 ± 3.4	35.5 ± 1.6	18.6 ± 1.7	3.6 ± 0.5	2.1 ± 0.3	0.35 ± 0.09	70.4 ± 6.1

**Table 3:** The serum biochemical parameters of quails in the experimental and control groups.

	Indicators							
	CRE mmol/L	AST ME/L	ALT ME/L	ALP ME/L	GLU mmol/L	CH mmol/L	TG g/L	TBIL mmol/L
5 days experiment	20.6 ± 0.5	2.1 ± 0.3	0.11 ± 0.02	3.7 ± 0.3	13.7 ± 0.4	4.3 ± 0.6	1.11 ± 0.14	1.6 ± 0.4
Control 5 days	20.4 ± 0.4	2.3 ± 0.3	0.07 ± 0.02	3.5 ± 0.4	14.2 ± 0.5	3.7 ± 0.4	0.99 ± 0.10	1.8 ± 0.4
10 days experiment	23.1 ± 0.3	2.7 ± 0.4	0.17 ± 0.03	5.3 ± 0.2	11.1 ± 0.4	2.8 ± 0.4	0.96 ± 0.12	2.3 ± 0.3
Control 10 days	18.8 ± 0.4	1.7 ± 0.3	0.11 ± 0.02	4.2 ± 0.3	12.3 ± 0.4	2.6 ± 0.4	1.2 ± 0.11	2.9 ± 0.6
15 days experiment	25.9 ± 0.3	2.5 ± 0.4	0.18 ± 0.02	5.9 ± 0.4	12.3 ± 0.4	2.5 ± 0.4	1.2 ± 0.11	2.5 ± 0.5
Control 15 days	17.9 ± 0.4	1.8 ± 0.3	0.09 ± 0.02	4.2 ± 0.3	12.6 ± 0.4	2.6 ± 0.4	1.2 ± 0.13	2.9 ± 0.5

**Table 4:** Number of microorganisms (lg) in 1 cm^3^ of quail feces.

Type of Microorganism	Number of Isolates					
	5 Days Experiment	Control 5 Days	10 Days Experiment	Control 10 Days	15 Days Experiment	Control 15 Days
*Candida* spp.	4.13 ± 0.21	6.12 ± 0.36	3.13 ± 0.24	6.27 ± 0.27	1.13 ± 0.16	5.88 ± 0.39
*Lactobacillus* spp.	6.28 ± 0.35	4.13 ± 0.25	6.43 ± 0.51	4.01 ± 0.32	5.89 ± 0.27	4.34 ± 0.31
*Bifidobacterium* spp.	5.67 ± 0.28	3.42 ± 0.36	5.51 ± 0.29	4.59 ± 0.41	5.81 ± 0.38	5.02 ± 0.25
*Streptococcus* spp.	2.61 ± 0.48	2.34 ± 0.40	2.27 ± 0.36	2.11 ± 0.40	2.33 ± 0.29	2.08 ± 0.35
*Staphylococcus* spp.	1.91 ± 0.35	2.47 ± 0.21	1.87 ± 0.31	2.70 ± 0.33	1.90 ± 0.37	1.71 ± 0.29
*Propionibacterium* spp.	1.65 ± 0.19	1.50 ± 0.11	2.10 ± 0.28	1.63 ± 0.22	1.73 ± 0.25	1.80 ± 0.19
*Escherichia* spp.	4.31 ± 0.22	3.97 ± 0.35	4.24 ± 0.40	4.00 ± 0.29	4.33 ± 0.32	4.37 ± 0.25
*Streptomyces* spp.	2.84 ± 0.23	3.14 ± 0.31	3.02 ± 0.19	2.94 ± 0.26	3.18 ± 0.20	3.41 ± 0.23
*Pasterella* spp.	2.59 ± 0.32	2.31 ± 0.44	2.63 ± 0.27	2.58 ± 0.39	2.11 ± 0.36	2.37 ± 0.40
*Bacteroides* spp.	1.47 ± 0.25	0.91 ± 0.30	1.23 ± 0.24	1.07 ± 0.19	1.25 ± 0.25	1.16 ± 0.22
*Proteus* spp.	2.54 ± 0.39	3.05 ± 0.24	2.78 ± 0.31	2.60 ± 0.27	3.10 ± 0.24	2.90 ± 0.29
*Bacillus* spp.	2.97 ± 0.13	2.97 ± 0.13	2.97 ± 0.13	2.97 ± 0.13	2.97 ± 0.13	2.97 ± 0.13
*Clostridium* spp.	0.93 ± 0.16	1.21 ± 0.20	1.33 ± 0.25	1.34 ± 0.28	1.51 ± 0.26	1.60 ± 0.21
*Eubacterium* spp.	2.68 ± 0.22	2.66 ± 0.24	2.74 ± 0.21	2.51 ± 0.30	2.70 ± 0.31	2.49 ± 0.25

## Data Availability

The data presented in this study are available on request from the corresponding author.
